# A Comprehensive Literature Review Discussing Diagnostic Challenges of Prinzmetal or Vasospastic Angina

**DOI:** 10.7759/cureus.83745

**Published:** 2025-05-08

**Authors:** Shruthi Aswathappa, Aolani J Watson, Bushra Nawaz, Abhishek Jani, Sara Razi, Srijana Baral, Gabriel W Chanayire, Rukhsana Hakeem, Anju George, Rabeeul Islam, Cheeranthodika Fahima, Ramsha Ali

**Affiliations:** 1 Medicine and Surgery, M.S. Ramaiah Medical College, Bengaluru, IND; 2 Medicine, Anhui Medical University, Hefei, CHN; 3 General Medicine, The Dudley Group NHS Foundation Trust, Dudley, GBR; 4 Medicine and Surgery, Islamic International Medical College, Rawalpindi, PAK; 5 Medicine and Surgery, Saint Louis University School of Medicine, Saint Louis, USA; 6 Medicine and Surgery, Gujarat Cancer Society (GCS) Medical College, Hospital and Research Centre, Ahmedabad, IND; 7 Medicine and Surgery, Islamic Azad University of Tehran, Tehran, IRN; 8 Medicine and Surgery, Gandaki Medical College, Pokhara, NPL; 9 Medicine and Surgery, All Saints University School of Medicine, Roseau, DMA; 10 Medicine and Surgery, Azad Jammu and Kashmir Medical College, Muzaffarabad, PAK; 11 Medicine and Surgery, Amala Institute of Medical Sciences, Thrissur, IND; 12 Medicine and Surgery, Calicut Medical College, Kozhikode, IND; 13 Medicine and Surgery, South West Medical University, Luzhou, CHN; 14 Medicine and Surgery, Peoples University of Medical and Health Sciences, Nawabshah, PAK

**Keywords:** angiography, diagnostic criteria, inflammatory biomarker, ischemia, non-obstructive, provocative testing, vasospastic angina

## Abstract

This narrative review addresses the diagnostic complexities of vasospastic angina (VSA), also known as Prinzmetal angina, by analyzing findings from peer-reviewed studies published over the past decade. It highlights clinical characteristics, guideline-directed approaches, and diagnostic strategies for VSA and ischemia with non-obstructive coronary arteries (INOCA). Although intracoronary provocation testing with acetylcholine or ergonovine remains the gold standard, its use is limited due to procedural variability and restricted access. Non-invasive modalities such as positron emission tomography (PET) imaging, cardiac magnetic resonance imaging (MRI), optical coherence tomography (OCT), and ambulatory electrocardiogram (ECG) monitoring have demonstrated promise but often lack consistency in diagnostic yield. Recent advances, including artificial intelligence (AI)-based ECG interpretation, inflammatory biomarkers, and microRNA profiling, are emerging as tools to improve diagnostic precision and risk stratification. VSA often remains underdiagnosed due to its transient symptoms, resemblance to acute coronary syndromes, and influence of patient factors like age, sex, comorbidities, and symptom variability. Enhancing diagnosis requires standardized testing protocols, broader use of coronary function testing, and integration of novel imaging and biomarker technologies. Recognizing atypical presentations, particularly in younger patients and women, is crucial to reducing misdiagnoses and improving clinical outcomes.

## Introduction and background

Vasospastic angina (VSA), or Prinzmetal angina, is characterized by transient coronary artery spasm leading to myocardial ischemia. Its episodic nature and absence of persistent arterial abnormalities pose a significant challenge to its diagnosis. This condition can occur in patients with both obstructive and non-obstructive coronary arteries, with transient spasm causing chest pain and persistent spasm potentially leading to acute myocardial infarction [1]. The unpredictable episodes of coronary vasospasm make it difficult to capture during standard diagnostic procedures. This makes specialized tests, such as provocative testing with agents like acetylcholine or ergonovine, necessary to induce spasm under controlled conditions. However, these provocative tests are not routinely performed due to potential adverse effects [2]. VSA can present with normal coronary angiograms, making it more complicated to differentiate it from other causes of chest pain, and requires an extensive evaluation. The pathogenesis involves complex mechanisms, including endothelial dysfunction and smooth muscle hypercontractility [3]. Accurate diagnosis is crucial, as VSA requires specific management strategies distinct from those for other forms of angina.

VSA remains a diagnostic challenge due to the limitations of current tools, including the risks of vasoreactivity testing [4]. Noninvasive markers, such as electrocardiogram (ECG) changes [5], intracoronary pressure variations [6], and genetic predisposition [7], have been explored, but their diagnostic value remains unclear. Corticosteroid-induced VSA complicates management [8], and distinguishing VSA from obstructive coronary artery disease (CAD) in acute coronary syndrome (ACS) remains critical [9]. Emerging imaging techniques [10], noninvasive indices [11], and interventional diagnostic procedures [12] may refine diagnosis. An unusual clinical presentation, mixed symptoms that overlap with other cardiac conditions, and variable responses to medical management suggest that this condition is underrecognized, making it difficult to manage [3,13]. International standardization of diagnostic criteria for VSA was given by the Coronary Vasomotion Disorders International Study (COVADIS) group in 2015, which helped to coordinate clinical and research work on VSA [14]. Some studies give insight that family history, smoking, female gender, elevated inflammatory biomarkers, autoantibodies, and genetic factors play an essential role in the development of VSA, which highlights the importance of investigation for appropriate diagnosis [15,16]. Further, the articles explain high mortality and morbidity with VSA, but initiation of calcium channel blockers improved overall results, whereas some patients develop recurrent vasospasm even after being treated adequately [17,18].

This review article aims to delve into the new diagnostic methods, from imaging to spastic provocation tests to biomarker testing, and the strengths, limitations, as well as the potential of emerging techniques and the role of artificial intelligence (AI) to ensure early detection and risk prediction of VSA and possibly prevent misdiagnosis of it as other non-cardiac chest pain.

## Review

Methodology

We conducted a comprehensive literature search to identify studies compatible with the diagnostic challenges of VSA.

Search Strategy

An extensive and structured search was initiated on PubMed in February 2025. The search terms that helped retrieve English-language publications were as follows: narrative reviews, systematic reviews, case reports, clinical trials, randomized controlled trials, and meta-analyses. PubMed using Medical Subject Headings (MeSH) search terms were 'vasospastic angina,' 'variant angina,' 'prinzmetal angina,' 'diagnosis,' and 'treatment,' and Boolean operators (AND, OR) were applied. We limited articles published within the last ten years to make our search centered on the most current research.

After the initial search, 650 studies on PubMed were found that met the above criteria. The Rayyan platform was used by three authors, who manually screened, analyzed, and removed duplicate publications from the titles, abstracts, and full texts.

Inclusion criteria: Studies included randomized controlled trials, narrative reviews, case reports, clinical trials, and meta-analyses published in English. To focus on the adult population, only participants aged 18 years and older were considered. Articles published between 2015 and 2025 were included to ensure the review reflects recent research. Only studies involving human subjects were considered.

Exclusion criteria: Studies published in languages other than English were excluded. Individuals under 18 years of age. Pregnant women were excluded because their management strategies are more likely to induce bias.

Data Extraction and Synthesis

After the screening, 213 articles were selected per our predefined inclusion and exclusion criteria, thus reducing the risk of selection bias. All authors independently collected data from relevant articles according to a structured plan, which included the following specifications: authors' names, digital object identifier (DOI), study type, year of publication, study objective, sample size, key findings, and relevance to diagnostic challenges of VSA. Consequently, all the extracted data was gathered in a Microsoft Excel (Microsoft Corporation, Redmond, Washington, United States) spreadsheet.

Discussion

Pathophysiology and Risk Factors

VSA has a complex pathophysiology that involves endothelial dysfunction, hypercontractility of vascular smooth muscle cells, and an increased presence of vasoconstrictive metabolites [3].

**Figure 1:**
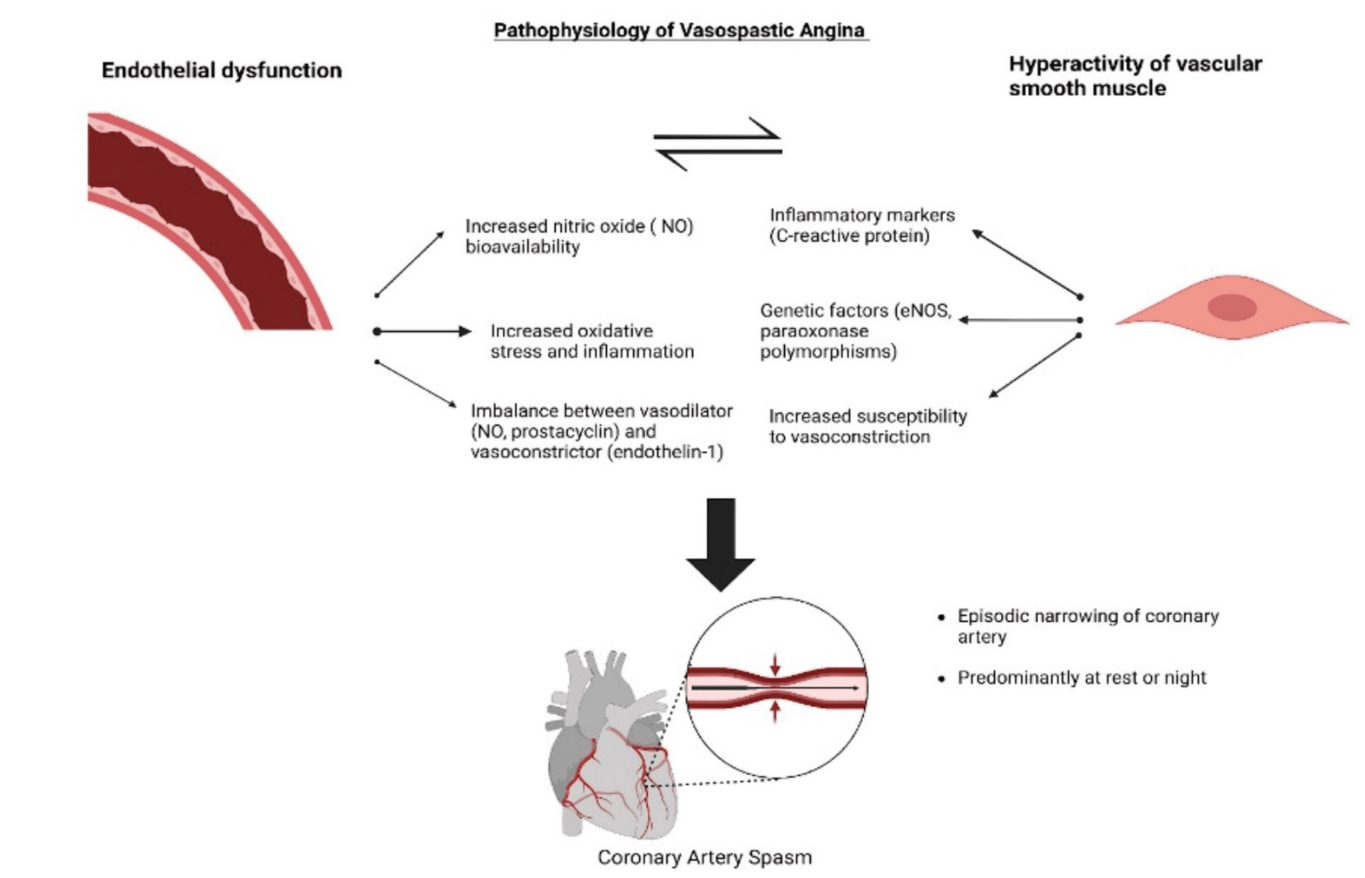
Pathophysiology of coronary vasospasm This image was generated by the author using BioRender (BioRender, Toronto, Canada) software. eNOS: endothelial nitric oxide synthase

Endothelium, the inner lining of blood vessels, maintains vascular tone by releasing various substances that regulate vasodilation and vasoconstriction. In a healthy state, the endothelium produces both vasodilators, such as nitric oxide (NO) and prostacyclin, which help relax vascular smooth muscle cells, as well as vasoconstrictors like endothelin-1 [3]. In the context of endothelial dysfunction, there is a reduction in NO bioavailability, leading to impaired vasodilation. This imbalance favors vasoconstriction, making the coronary arteries more susceptible to spasms. Additionally, endothelial dysfunction is associated with increased oxidative stress and inflammation, which further impair endothelial function and contribute to the hyperreactivity of vascular smooth muscle cells (Figure 1) [19].

There are also thought to be genetic risk factors associated with coronary artery spasm; these include gene polymorphisms of endothelial NO synthase, paraoxonase, and other genes.

VSA angina presents significant variations across different populations, influenced by genetic, environmental, and demographic factors. Genetic predisposition plays a crucial role, with mutations in genes like endothelial nitric oxide synthase (eNOS) and human leucocyte antigen (HLA) abnormalities contributing to familial cases. Monozygotic twins and sibling studies highlight an inherited component, making family history an essential factor in diagnosis. Notably, the RNF213 p.R4810K variant, found in 1.5% to 2.5% of Japanese and Koreans, is strongly linked to VSA and moyamoya disease, particularly in women and those with dyslipidemia, suggesting a shared mechanism involving NO regulation [7,20,21,22].

Ethnicity significantly affects VSA prevalence and presentation, with East Asian individuals, especially Japanese and Korean individuals, showing a higher incidence. These populations often develop VSA without traditional CAD risk factors, necessitating a different diagnostic approach. Japanese patients are predominantly male, heavy smokers, and frequently experience nighttime angina. Their treatment strategies also differ, with 96% receiving calcium-channel blockers, compared to Caucasian individuals, who more commonly use nitrates (59%), statins (65%), and β-blockers (24%) [23]. Regional disparities also impact diagnostic accuracy. In Australia and New Zealand, inconsistent functional coronary angiography (FCA) protocols and varied acetylcholine dosing contribute to underdiagnosis, which limits effective treatment strategies [24].

Environmental factors further shape VSA risk and presentation. Chronic exposure to particulate air pollution (PM10) significantly increases the frequency of coronary artery spasms (CAS) and associated ST-segment changes. Studies show that a 20 µg/m³ rise in PM10 levels correlates with a 4% increase in CAS risk, highlighting the role of pollution as a modifiable risk factor [25].

Gender differences in VSA presentation and prognosis are striking. Men are diagnosed at a younger age, often have higher rates of smoking and alcohol consumption, and exhibit more organic coronary stenosis, leading to worse initial cardiovascular outcomes. Their ECG findings more frequently show ST-segment elevation, while women exhibit more T-wave inversion, a pattern that is not yet fully understood. In contrast, women under 60 tend to have fewer atherosclerotic lesions but require higher doses of ergonovine during provocative testing. They experience symptoms more frequently at night, with spasms commonly occurring in the right coronary artery, while men have more left circumflex artery involvement. Interestingly, while high-sensitivity C-reactive protein (hs-CRP) predicts poor outcomes in men, it is not a significant prognostic marker in women. A higher BMI has been associated with better outcomes in female patients, suggesting that there are gender-specific protective factors [26,27,28].

Autonomic nervous system imbalance, characterized by heightened sympathetic activity and parasympathetic withdrawal, plays a crucial role in the pathogenesis of VSA. Studies show VSA patients exhibit significantly higher muscle sympathetic nerve activity (MSNA), with an increase from 56.8 bursts/min to 66.1 bursts/min under mental stress. Comorbidities like hypertension (40%) and diabetes (7%) contribute to endothelial dysfunction, heightening the likelihood of coronary spasm. Additionally, smoking, which affects 40% of VSA patients, worsens vascular tone and impairs vasoregulation. These factors emphasize the need to manage autonomic dysregulation and comorbidities to reduce the risk of VSA [29].

Hyperthyroidism increases VSA risk by enhancing vasomotor activity and making coronary arteries more sensitive to vasoconstriction, particularly in women. Thyroid function testing is included in the diagnostic approach for patients with chest pain and suspected VSA. However, hyperthyroid patients tend to have a prognosis similar to non-hyperthyroid VSA patients [30]. Asthma, especially inhaled corticosteroid use, also raises the risk of VSA, influencing clinical presentation and diagnostic strategies [31].

Smoking is a significant risk factor for VSA, significantly increasing recurrent angina rates (HR 2.46, 95% CI 1.46-4.14) by worsening endothelial dysfunction and triggering coronary spasms [32]. It impairs vasoregulation, reduces NO, and promotes smooth muscle hyperreactivity. Notably, sudden smoking cessation may trigger VSA due to the loss of nicotine's protective effect on the parasympathetic system [33].

Overweight and obesity in VSA patients have been associated with a lower rate of major adverse events (cardiac death, new arrhythmia, and ACS), supporting the "obesity paradox" observed in CAD [34]. However, dyslipidemia remains a significant risk factor for worse outcomes. In diabetic patients, endothelial dysfunction and atherosclerosis worsen VSA, with a higher prevalence of diffuse coronary spasms. While major adverse cardiovascular event (MACE) rates do not differ significantly, diabetic patients with focal spasms have reduced MACE-free survival, highlighting the need for tailored management [35]. Additionally, the use of sodium-glucose transporter-2 inhibitors (SGLT2i) in diabetes has been linked to euglycemic diabetic ketoacidosis (eDKA), which can trigger severe coronary vasospasm, emphasizing the importance of careful monitoring [36].

VSA is also associated with various comorbidities, complicating diagnosis and management. Spontaneous coronary artery dissection (SCAD) shares common risk factors with VSA, including middle age, emotional stress, and endothelial dysfunction. In some cases, SCAD presents with coexisting VSA, identified through intracoronary imaging and vasoreactivity testing. In these instances, calcium channel blockers may be more effective than β-blockers, which could worsen vasospasm [37]. VSA is linked to myocarditis, with elevated plasma cytokine levels and increased myocardial inflammation observed in VSA patients, necessitating inflammation-focused evaluations alongside coronary angiography [16].

Kawasaki disease can contribute to long-term coronary endothelial dysfunction, causing both microvascular and epicardial VSA. In these patients, vasoreactivity testing may be required to confirm ischemia with non-obstructive coronary arteries (INOCA) [38]. Granulomatosis with polyangiitis (GPA) can present with VSA, though it is often underrecognized due to the lack of significant stenosis on coronary angiography. Multimodal imaging, such as PET-CT, can help identify GPA-related VSA [39].

Long QT syndrome (LQTS) can coexist with VSA, complicating treatment since β-blockers commonly used for LQTS may exacerbate vasospasm, increasing the risk of arrhythmias [40]. Takotsubo cardiomyopathy (TCM), especially during stress-induced catecholamine surges, can trigger vasospasm, complicating diagnosis and management when multivessel vasospasm is present [41].

COVID-19 may allegedly contribute to VSA by causing endothelial dysfunction and initiating inflammatory responses, making coronary arteries more sensitive to vasoconstriction. This heightened sensitivity can trigger or worsen VSA, particularly during recovery [42]. Similarly, the mRNA COVID-19 vaccines may also trigger VSA through ACE2 downregulation, the activation of inflammatory cytokines, and potential allergic reactions, which can increase the risk of vasospasms. Genetic predisposition, lipid nanoparticles, and Kounis syndrome could further elevate VSA risk following vaccination [43].

Diagnostic Methods

Patients with VSA report a broad spectrum of symptoms, from mild discomfort to severe chest pain indistinguishable from ACS. Some experience lengthy anginal episodes, while others have brief, recurrent attacks. Additionally, symptoms may extend beyond chest pain, presenting as jaw, arm, or back discomfort; syncope or near-syncope; palpitations; cold sweats; and nausea [44,45,46]. The inconsistency in symptom presentation contributes to under-recognition, particularly in younger patients or those without conventional cardiovascular risk factors such as hypertension, diabetes, or smoking history [35,47,48].

Standard diagnostic tests often fail to identify VSA, leading to missed or delayed diagnoses. While ECG, troponin levels, stress testing, and conventional coronary angiography are essential in evaluating chest pain, they may not reliably confirm VSA. ECG abnormalities in VSA are transient and often return to normal between episodes, making the ECG an unreliable standalone diagnostic tool that usually resolves before the patient seeks medical attention.

Many patients with transient ECG changes undergo emergency coronary angiography, only to reveal INOCA, leading to diagnostic uncertainty. This can be seen in a case report detailing a 69-year-old female who presented with clinical features and ECG changes consistent with ST elevation myocardial infarction (STEMI). The coronary evaluation revealed findings consistent with VSA rather than obstructive CAD [49].

Additionally, VSA may be misclassified as unstable angina or non-ST elevation MI (NSTEMI) when symptoms occur sporadically without definitive ECG abnormalities. The transient nature of coronary spasm means that by the time a patient arrives at the emergency department, ECG changes may have resolved, further complicating diagnosis. This can be seen in the case of VSA, which was diagnosed by a personal handheld ECG device [50].

Advanced ECG monitoring provides critical insights into transient ischemic events underlying the condition. During an angina episode, transient ischemic changes, such as ST elevation or depression, may be observed. This may resolve once the spasm subsides. Ambulatory ECG monitoring enables continuous recording over 24-48 hours and helps capture ischemic events. During vasospastic episodes, ST-segment elevation or depression, a negative U wave, or P wave inversion can be seen, as well as ventricular tachycardia. In patients with symptoms of suspicious VSA, every effort should be undertaken to obtain an ECG during spontaneous episodes of VSA. Then, there should be a low threshold for performing invasive coronary functional testing (ICFT) [5,51,52].

Coronary angiography with provocative spasm testing is the gold standard for diagnosing VSA. This involves the administration of vasoactive agents such as acetylcholine, ergonovine, or methylergonovine to induce spasm under controlled conditions. However, protocols vary across institutions [19,53].

Acetylcholine-induced spasm tends to be diffuse and distal, while ergonovine-induced spasm is focal and proximal. Both tests have limitations in reproducing daily-life coronary spasms, and combining them can improve diagnostic accuracy. Acetylcholine is particularly sensitive in women [2].

There is an increased rate of major adverse cardiac events in patients with positive ergonovine echocardiographic (ErgECHO) tests. VSA patients confirmed by ErgECHO have a higher incidence of MACE over long-term follow-up [54].

It is well known that there is provocative testing variability. A 2016 case report showed that three out of four patients (75%) had a positive second spasm provocation test after the initial test was negative [55]. The reproducibility of provocation testing remains a concern. Some patients exhibit an intermediate response that is neither fully diagnostic nor entirely normal, which complicates interpretation and creates ambiguity in clinical decision-making. Long-term follow-up data are absent for these patients. Furthermore, reproducibility remains an issue, as daily variations in vasoreactivity may lead to underestimation or overestimation of disease severity [4].

Recent studies suggest a dose-dependent relationship between acetylcholine provocation and anginal symptoms. A significant observational study in 2025 showed that 509 patients with angina with non-obstructive coronary arteries (ANOCA) indicated an inverse correlation between the acetylcholine dose required to induce spasm and symptom severity, suggesting its potential utility as a prognostic risk stratification tool [56].

In the United States, ergonovine is not readily available, and acetylcholine testing is limited to specialized centers, limiting its use in routine practice [57].

A case study in 2016 showed that a 72-year-old female patient with suspected coronary vasospasm and poor symptom control underwent an OCT during an ergonovine test. The ergonovine test was positive, and the OCT showed an underlying atherosclerotic disease, which was not detected by coronary angiography. OCT is useful for establishing the initial pathophysiological process of VSA in patients; however, it currently has a limited role in evaluating VSA and would require further studies [58].

OCT helps in the detailed visualization of the coronary artery wall. OCT can detect rare structural changes, such as internal tears or detachment, that may mimic VSA symptoms, ensuring accurate diagnosis. It helps in assessing endothelial dysfunction. It can detect irregularities in endothelial integrity that may not be visible on angiography. OCT can monitor vascular healing and the response to therapies, such as calcium channel blockers (CCBs) and nitrates. For patients undergoing treatment, OCT can evaluate changes in the coronary lumen during adenosine, acetylcholine, or ergonovine provocation, which helps confirm the diagnosis of VSA. By assessing plaque morphology, OCT can differentiate between vasospasm-induced narrowing and atherosclerotic plaque stenosis. OCT can determine the microvascular network for abnormality, providing evidence of microvascular spasms that may contribute to VSA symptoms [59].

Without substantial angiographic disease, intravascular ultrasound (IVUS) can recognize plaque composition and intimal hyperplasia at the site of artery spasm. In juxtaposition, OCT can accurately demarcate structural modifications in spastic coronary arteries [3].

IVUS provides a detailed insight into the structure and pathology of the coronary artery. It allows us to create a cross-sectional image of coronary artery walls. It helps identify structural changes, such as thickening and fibrosis associated with VSA. It reveals even subtle changes in the endothelial layer that may not be visible in coronary angiography. It helps to differentiate between vasospasm-induced narrowing and stenosis related to atherosclerotic plaque. IVUS can be used in conjunction with provocation to assess changes in vessel diameter and wall structure during and after a spasm episode [60].

Coronary flow reserve (CFR) and Index of Microcirculatory Resistance (IMR) are used to assess microvascular dysfunction, which often coexists with VSA [61,62]. Fractional flow reserve (FFR) helps evaluate the hemodynamic significance of coronary lesions, particularly in cases of suspected INOCA [62]. Intracoronary pressure measurements may help predict coronary spasms, although further evidence is required [6]. Non-invasive assessments that include myocardial blood flow reserve (MBFR) measurements using stress PET or stress cardiac magnetic resonance (CMR) imaging can detect coronary microvascular dysfunction (CMD) and enhance risk stratification in patients with suspected INOCA. These diagnostic tests have low accuracy for diagnosing vasomotor dysfunction, with a specificity and sensitivity of 57% and 41%, respectively [63].

While invasive testing is the ultimate approach, non-invasive methods have been studied to improve VSA detection. Blood tests, including C-reactive protein (CRP), lipid profile, and hemoglobin A1c, can be used to identify systemic vascular risk factors. Therefore, non-invasive assessments primarily focus on identifying CMD as a cause of angina. Troponin levels are unreliable in diagnosing VSA. Unlike myocardial infarction, which causes persistent troponin elevation, VSA does not always lead to detectable troponin release unless vasospasm results in prolonged ischemia [64].

Stress testing by exercise often fails to induce vasospasm, as VSA occurs predominantly at rest or in response to specific triggers, such as hyperventilation or cold exposure [36,65]. Standard coronary angiography may help, but notably, many patients with VSA have no significant coronary stenosis, and routine angiography without functional testing may not reveal vasospasm [66].

Ergonovine stress echocardiography can be performed safely by experienced clinicians and may serve as an alternative to invasive coronary angiography for diagnosing VSA. A positive result has also been linked to long-term adverse cardiovascular outcomes [54].

A study by Yang et al. used an ex vivo biologic approach utilizing peripheral blood. It demonstrated that the different responses in intracellular calcium efflux of vascular smooth muscle cells (VSMCs) could represent a diagnostic marker [67]. Serum biomarkers such as malondialdehyde-modified low-density lipoprotein (MDA-LDL) have been proposed as a potential biomarker [68].

Inflammatory markers can be helpful, as inflammation plays a crucial role in the development of coronary spasm. Pro-inflammatory cytokines have also been studied for their potential diagnostic capabilities [56]. The pro-inflammatory cytokines IL-4, interferon-α, IL-1α, IL-2, and the chemokine monocyte chemoattractant protein-1 are significantly higher in patients with VSA than in those with acute myocardial injury [16]. A clinical study suggested a diagnostic and pathophysiological role of circulating microRNA (miR) in regulating eNOS expression in human coronary artery endothelial cells (hCAECs) [69]. This process is correlated to endothelial impairment or smooth muscle cell pathology, which could cause VA. The study showed that patients diagnosed with VSA exhibited increased expression levels of these miR. The miR profiles in serum circulation could represent potential novel biomarkers for VSA diagnosis.

Myocardial scintigraphy methods help diagnose patients with VSA. While hyperventilation testing and metaiodobenzylguanidine (MIBG) scintigraphy were once used for VSA diagnosis, recent trends suggest that cardiologists should consider thallium (Tl) or beta-methyl-p-[123I]-iodophenyl-pentadecanoic acid (BMIPP) myocardial scintigraphy as supplementary tools, as they have higher sensitivity and specificity [70]. PET and CMR are imaging modalities that can be used to assess myocardial perfusion and detect occult myocardial infarction in patients with ANOCA. Still, their role in routine VSA diagnosis remains uncertain [71]. Non-invasive imaging tools such as fluorine-18 fluorodeoxyglucose positron emission tomography (18F-FDG PET) are used to detect vascular inflammation. An advanced inflammatory imaging modality, PET/CT with gallium-68-labeled DOTATATE (68Ga-DOTATATE), has been demonstrated to provide superior image quality compared to 18F-FDG PET/CT [72].

Imaging used in VSA helps us to assess myocardial perfusion and structural abnormalities. MRI techniques, such as perfusion imaging and coronary MRI, can assess myocardial blood flow. They can detect regions of the heart muscle that are not receiving adequate blood supply due to blocked or narrowed coronary arteries. It provides insight into the composition and stability of atherosclerotic plaque, such as whether a plaque is soft, calcified, or fibrous. It also helps measure coronary blood reserve. A decreased CFR can indicate impaired function of the coronary arteries. Coronary MRI is a non-invasive procedure that does not even require radiation [1]. A comparison of these various diagnostic tests is shown in Table 1.

**Table 1:** Comparison of various non-invasive and invasive diagnostic methods VSMC: vascular smooth muscle cell; MDA-LDL: malondialdehyde-modified low-density lipoprotein; VSA: vasospastic angina

Diagnostic Method	Type	Pros	Cons
Electrocardiography (ECG)	Non-invasive	Readily available, low-cost, detects transient ischemic changes [49]	Changes are often transient and resolve before medical evaluation [49,50]
Troponin Levels	Non-invasive	Helps rule out myocardial infarction [64]	Unreliable for VSA unless prolonged ischemia occurs [64]
Stress Testing (Exercise/Pharmacologic)	Non-invasive	Can assess ischemia under stress conditions [36,65]	Often fails to induce vasospasm since VSA occurs mainly at rest [36,65]
Standard Coronary Angiography	Invasive	Can rule out obstructive coronary artery disease [66]	May miss dynamic coronary spasm unless functional testing is done [66]
Coronary Angiography With Provocation Testing	Invasive	The gold standard for diagnosing VSA [19,53]	Limited availability, variability in protocols, and reproducibility issues [19,53,57]
Optical Coherence Tomography (OCT)	Non-invasive	Detects microstructural artery changes during spasm [59]	Limited data on its use for VSA requires further studies [58]
Intravascular Ultrasound (IVUS)	Invasive	Identifies plaque composition, can detect intimal hyperplasia [60]	It does not directly assess functional vasospasm [60]
Coronary Flow Reserve (CFR) and Index of Microcirculatory Resistance (IMR)	Invasive	Assesses microvascular function [61,62]	Requires specialized equipment and expertise [61,62]
Fractional Flow Reserve (FFR)	Invasive	Evaluate the hemodynamic significance of coronary lesions [62]	Limited role in detecting VSA-related spasms [62]
Ergonovine Stress Echocardiography (ErgECHO)	Non-invasive	Alternative to invasive testing may indicate long-term risk [54]	Requires experienced operators, limited sensitivity [54]
Peripheral Blood VSMC Calcium Efflux Test	Non-invasive	A potential diagnostic marker for VSA [67]	Still in the experimental stages [67]
Serum Biomarkers (MDA-LDL, Cytokines, MicroRNAs)	Non-invasive	May help identify the inflammatory role in VSA [16,68,69]	No standardized clinical application yet
Myocardial Scintigraphy	Non-invasive	Assesses myocardial perfusion and ischemia [70]	Expensive, not widely available for routine use [70]
Cardiac Magnetic Resonance (CMR)	Non-invasive	Can detect subtle myocardial changes [71]	Limited role in routine VSA diagnosis [71]

Diagnostic Criteria

**Figure 2:**
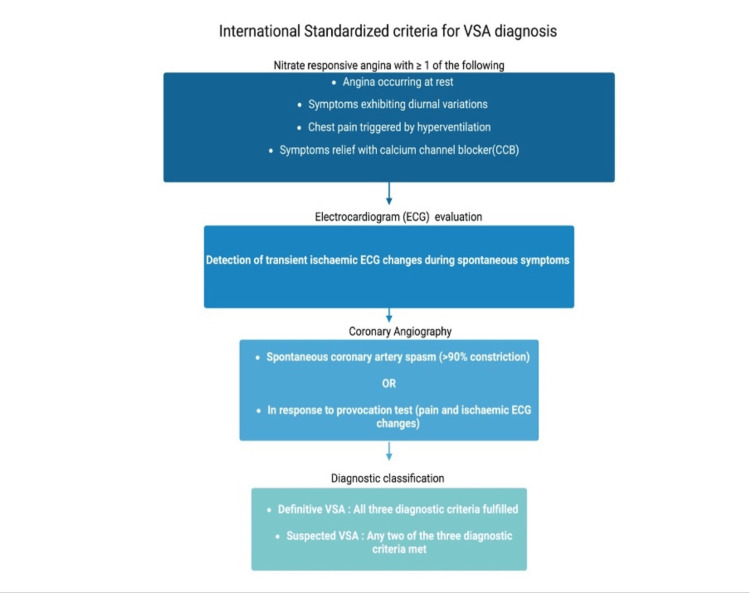
International standardization criteria for VSA diagnosis This image was created by the author using the Biorender (BioRender, Toronto, Canada) software. VSA: vasospastic angina

A more well-known criterion is the internationally standardized one established by the COVADIS group. For a definitive diagnosis of VSA, the criteria required are nitrate-responsive angina symptoms with at least one of the following: rest angina, diurnal variation in symptoms, hyperventilation-induced angina, or symptom improvement with calcium-channel blockers (CCBs); transient ischemic ECG changes during spontaneous symptoms; documented coronary artery spasm (>90% constriction) occurring spontaneously or in response to provocation testing (with pain and ischemic ECG changes). If only two of these criteria are met, the patient is diagnosed with 'suspected VSA' (Figure 2) [1,73].

Various international organizations have developed different sets of guidelines for diagnosing VSA. The comparison between the European Society of Cardiology (ESC), the American College of Cardiology (ACC), the Japanese Circulation Society (JCS), and the COVADIS is presented in Table 2.

**Table 2:** Summarizing the different guidelines by JCS, ESC, ACC, and COVADIS

Criteria	Japanese Circulation Society (JCS)	European Society of Cardiology (ESC)	American College of Cardiology (ACC)	Coronary Vasomotion Disorders International Study Group (COVADIS)
Primary Diagnostic Approach	Invasive coronary angiography with provocation testing (acetylcholine/ergonovine) [62]	Clinical symptoms + transient ischemic ECG changes ± provocative testing [74]	Clinical symptoms + transient ST elevation ± provocative testing [75]	Requires ≥2 of: nitrate-responsive angina, transient ischemic ECG changes, and spontaneous or provoked coronary spasm [76]
Role of Provocative Testing	Strongly recommended with defined protocols [62]	Acknowledged, but not mandatory [74]	Recommended when non-invasive tests are inconclusive [75]	Emphasized for standardized diagnosis, but no specific protocols [76]
ECG Criteria	ST-segment elevation or depression during an angina episode [62]	Transient ST-segment changes (elevation or depression [74]	Transient ST-segment elevation during episodes [75]	Transient ischemic ECG changes required for diagnosis [76]
Angiographic Findings	Total or subtotal coronary artery narrowing during provocation [62]	Optional but recommended in uncertain cases [74]	Optional, recommended in specialized centers [75]	Spasm must be evident on angiography if using provocation testing [76]
Risk Stratification	Based on location/extent of spasm, with specific recommendations [62]	Included within broader ischemic heart disease guidelines [74]	Considered within general angina guidelines [75]	Not the main focus, but acknowledges risk assessment [76]
Management Guidelines	Calcium channel blockers & nitrates as first-line therapy [62]	Calcium channel blockers & nitrates + lifestyle modifications [74]	Calcium channel blockers & nitrates, risk factor modification [75]	Focus on medical therapy, particularly calcium channel blockers & nitrates [76]

Diagnosis

ESC guidelines: The role of provocation testing is emphasized, but the role of non-invasive assessments, such as extended Holter monitoring, is also considered. It uses a pre-test probability model incorporating factors such as age, sex, and symptoms. States VSA can be diagnosed based on spontaneous episodes of angina at rest with transient ischemic ECG changes, even without angiographic documentation of spasm [74].

ACC guidelines: Considers both invasive and non-invasive diagnostic approaches. Recommends provocative testing when initial non-invasive tests are inconclusive and highlights the clinical presentation of rest angina with transient ST-segment elevation [75].

JCS guidelines: Mainly focuses on invasive coronary angiography with pharmacological provocation tests using either acetylcholine or ergonovine to induce and document coronary spasms. A positive test is considered when there is transient, total, or subtotal coronary artery narrowing along with chest pain and ischemic ECG changes [62].

COVADIS criteria: The COVADIS group established standardized diagnostic criteria that require nitrate-responsive angina, transient ischemic ECG changes, and spontaneous or provoked CAS. Definitive diagnosis is based on at least two of these positive components [76].

Provocative Testing Protocols

ESC and ACC guidelines: The utility of provocative testing is acknowledged, but it does not provide any specific protocols. Recommends that provocative tests be used in specialized centers with experts trained in invasive coronary procedures [74,75].

JCS guidelines: The primary focus is on provocation testing using intracoronary administration of acetylcholine and ergonovine, which provides a detailed protocol for specifying dosages and interpreting responses to assess for coronary spasm [62].

COVADIS criteria: It emphasizes the importance of standardized provocative testing to ensure consistent diagnosis across different centers, but does not prescribe specific testing protocols [76].

Risk Stratification and Management

ESC and ACC guidelines: VSA is considered part of the spectrum of ischemic heart diseases while providing broader management strategies for angina. They recommend CCBs and nitrates, as well as lifestyle modifications and risk factor management, as integral components of therapy [74,75].

JCS guidelines: There are detailed recommendations on risk stratification based on the patient's symptoms and the extent and location of the spasm. Management strategies emphasize CCBs and nitrates as first-line therapies [62].

COVADIS criteria: While primarily focused on diagnostic standardization, COVADIS acknowledges the importance of appropriate medical therapy, mainly CCBs and nitrates, in managing VSA [76].

In summary, the JCS provides specific protocols for testing, whereas the ESC and ACC recommend their use in specialized settings without detailed protocols. COVADIS focuses on standardizing diagnostic criteria to facilitate consistent diagnosis across various clinical settings. There is consensus on the clinical presentation and primary treatment options for VSA; differences exist in the emphasis on diagnostic approaches, particularly regarding the role and standardization of provocative testing [62,74,75,76,77].

Future research in diagnostic approaches to VSA should focus on the standardization of invasive provocation testing protocols, development of newer diagnostic methods such as IVUS and OCT, exploration of genetic and environmental factors, investigation of endothelial dysfunction and smooth muscle hyperreactivity, identification of blood-based biomarkers such as endothelial dysfunction markers and inflammatory cytokines, and development of wearable devices capable of real-time monitoring of cardiovascular parameters.

AI is making strides as a valuable tool in helping clinicians make decisions related to multiple aspects of clinical practice, including cardiology [78,79]. The use of AI in diagnosing VSA is based on its ability to analyze the ECG. A study demonstrated that AI-enabled algorithms could rule out CMD in certain patients. CMD was associated with minor ECG differences (QTc and T waves) [80], which AI models can detect. Confirming the CMD diagnosis varies by center and requires specialized tools and methods. As a result, an algorithm integrating demographic and ECG data would offer significant value. However, AI algorithms could not distinguish between patients with CMD and those without CMD with high sensitivity and specificity [81]. AI helps optimize provocation tests by predicting which agents will yield the most diagnostic value based on patient-specific factors [82,83]. There are technical challenges when attempting to incorporate AI algorithms into clinical practice.

An example is data standardization. Variations in existing ECG input data types, storage formats, and interpretation statements unavoidably limit the broad interoperability of ECG data. Another concern is cybersecurity, as AI algorithms can be a potential target for hacking. Almost all stages of the AI architecture's processing, including initial data inputs and human-machine teaming, as well as the data conditioning process, are susceptible to cyberattacks [84]. Considering all these, more studies are needed to extend the validity of using AI as a diagnostic tool and its widespread acceptance.

Diagnostic challenges

There are many studies highlighting challenges in the diagnosis of coronary vasospasm. We have divided these factors into two main categories.

Patient/Disease State Factors

Coronary vasospasm is a spectrum of disease varying from mild to severe spasms, leading to clinical presentations and complications very similar to obstructive CAD. Mild to moderate disease may remain undetected or asymptomatic; the sensitivity of vasoreactivity testing in patients with low to moderate disease is not always satisfactory [4]. There may also be negative vasoreactivity test results in some patients with strongly suspected VSA. This misdiagnosis may be related to disease activity, the residual effect of coronary vasodilators, the circadian variation of the disease, or imperfect standard vasoreactivity testing [4]. Clinical presentation can vary between patients due to different thresholds for ischemic pain and variations in circadian disease [4]. Inside the catheterization labs, when performing provocative testing, it is essential to consider that patients' pain responses are highly variable, depending on their threshold for pain stimuli.

Provocative Procedure Factors

Provocative agent (acetylcholine, ergonovine): The JCS, COVADIS group recommends using acetylcholine and ergonovine as vasoactive agents to diagnose VSA. Acetylcholine acts on muscarinic receptors, while ergonovine acts on serotonergic receptors, as their receptors are different. There may be variability in the effect of the two in even the same patients. Patient gender also responds differently to Ach; i.e., females tend to be more sensitive than males, whereas males react differently to ergonovine [4].

Dose of spasm-provocative agent: The COVADIS group recommends using a maximum dose of acetylcholine for the right coronary artery (RCA) and left coronary artery (LCA) of 50 and 100 mg, respectively, and has no recommendations for ergonovine doses. JCS guidelines recommend administering 40-60 μg of ergonovine in each coronary artery for a few minutes [4]. These differences in guidelines and the use of different doses of provocative agents in different ethnicities may produce high variability of results. The clear guidelines on the maximum dose of pharmacological agents used in provocative testing need to be refined to be universally accepted.

Selection of the RCA or LCA for testing: Vasospasms of the coronary artery have been observed in all three major vessels of the heart. Some physicians prefer testing in LCA, while many prefer testing both arteries, as testing a single artery may lead to a failed diagnosis [4,85]. Using both coronary arteries in a provocation test can yield a high number of positive cases and, hence, should be included in guidelines.

Cardiologists should consider the restrictions mentioned above while performing provocative testing in catheterization labs. To formulate a standard guideline for diagnostic testing in VSA, it is necessary to consider the issues highlighted above, including different populations, ethnicities, genders, genetic variation, and various vasospastic triggers, such as non-pharmacological agents like cold exposure.

The episodic and unpredictable nature of VSA is one of the significant factors that may lead to physicians missing the diagnosis of Prinzmetal angina. Symptoms occur intermittently, often at rest or in response to specific triggers, while patients remain asymptomatic between episodes, making it challenging to gather diagnostic evidence for research purposes [1,86]. Furthermore, due to symptom and trigger variability among patients, it is challenging to establish standardized diagnostic criteria. Atypical symptoms, such as chest pain without classic ST-segment elevation and silent ischemia, coexisting medical conditions (e.g., hypertension, diabetes mellitus), and a genetic predisposition to vascular disorders (eNOS, Rho-kinase, and other genes) make it difficult for researchers to establish well-structured diagnostic criteria for VSA [87].

Many patients who experience VSA might have other medical conditions and take many various medications that manipulate symptoms and test results. CCBs, nitrates, and statins can reduce spasm frequency. Alcohol use, smoking, or any lifestyle habits like regular cardio exercise introduce confounding factors, making results more complex to interpret [87,88].

Additionally, age- and sex-related variations make it challenging to apply a one-size-fits-all diagnostic approach. For example, women with VSA may present with more diffuse pain or have hormonal influences on vascular tone. Younger patients tend to have more active vasospastic responses, while older patients may have mixed ischemic patterns [89,90].

Limitations

Research on VSA diagnosis faces several challenges that impact its accuracy, reliability, and applicability in clinical practice. The limited sensitivity and specificity of diagnostic tests, such as acetylcholine or ergonovine provocation tests or ambulatory ECG monitoring, may lead to misdiagnosis, misclassification, or even overdiagnosis of VSA. Some studies avoid provocation testing due to the risk of severe coronary spasms or arrhythmias and rely on indirect diagnostic methods, which reduce accuracy. Furthermore, studies show VSA is more prevalent in East Asian individuals compared to Western populations, suggesting genetic or environmental influences, so most of the existing VSA studies have been conducted on small cohorts and often limited to a single medical center where VSA prevalence is higher, leading to ethnic and geographic bias. This leads to results that are not generalizable to broader populations [86,88,89].

Different guidelines and definitions of positive-interpreted provocation test results across countries, along with heterogeneous clinical presentation, make it challenging to establish standardized diagnostic criteria, compare study results, or apply research findings uniformly in clinical settings. Moreover, the lack of follow-up data makes it difficult to assess disease progression, treatment response, or the risk of complications such as sudden cardiac death [48]. Advanced testing for VSA is limited to optical coherence tomography and endothelial biomarker testing, which makes it challenging to improve diagnostic accuracy.

## Conclusions

VSA, also known as Prinzmetal angina, remains a challenging condition to diagnose due to its episodic nature and the variability in sensitivity and specificity of diagnostic tests. While advancements have been made, no single method provides a universally accepted standard for accurate detection. Despite its limitations, provocative spasm testing is crucial in confirming VSA. However, variability in testing responses can lead to ambiguity in clinical decision-making. Other diagnostic measures, such as CFR, IMR, and FFR, contribute to evaluating INOCA. Future progress in VSA diagnosis and management requires a more unified and structured approach. More extensive, diverse studies are needed to refine diagnostic protocols and establish standardized testing criteria. Integrating emerging technologies, such as advanced imaging and AI, holds promise for enhancing detection accuracy. Additionally, fostering collaboration among researchers and clinicians, as well as increasing awareness among healthcare providers, will be crucial in improving both diagnosis and patient care. Addressing these challenges will enhance medical accuracy and directly improve the quality of life for individuals affected by this often-overlooked condition.
